# Microbiome alterations and their potential impact on infection risk in chronic kidney disease and end-stage kidney disease: a narrative review

**DOI:** 10.1007/s11255-026-05061-z

**Published:** 2026-03-04

**Authors:** Lucia Federica Stefanelli, Leda Cattarin, Francesca Katiana Martino, Dorella Del Prete, Buscaglion Pierantonio, Federico Nalesso, Maria Mazzitelli

**Affiliations:** 1 Department of Medicine, University of Padua, Padua, Italy; 2 Unit of Nephrology, Dialysis and Transplantation, Padua University Hospital, Padua, Italy; 3 Medical University of Varna, Varna University Hospital, 9002 Varna, Bulgaria; 4 Università Cattolica Del Sacro Cuore, Department of Safety and Bioethics, Section of Infectious Diseases, 00168 Rome, Italy; 5 A. Gemelli University Hospital IRCCS Foundation, Department of Medical and Surgical Sciences, 00168 Rome, Italy

**Keywords:** Microbiome, Infections, Chronic kidney disease, Dysbiosis

## Abstract

Chronic kidney disease [CKD] is associated with profound alterations of both gut microbiota composition and functions, commonly referred to as gut dysbiosis. These changes are driven by several factors such as dietary restrictions, medications, and uremia, which further contribute to patients with CKD to their pro-inflammatory and immunocompromised state. Recent evidence suggests that dysbiosis may also increase susceptibility to infections, which remain a leading cause of morbidity and mortality in CKD patients. In this review, we aimed at examining the features of the altered gut microbiome in patients with CKD, its potential role in promoting infection risk, and current therapeutic strategies targeting the gut microbiota to mitigate CKD-related complications.

## Introduction

Chronic kidney disease [CKD] represents a major global health issue, affecting more than 10% of the adult population worldwide [1]. Its prevalence has steadily increased over the past decades, a trend largely attributable to the prolonged survival of individuals at risk for kidney dysfunction—such as those with diabetes, hypertension, or cardiovascular disease—and to the progressive aging of the population, often associated with multimorbidity and chronic, debilitating conditions [1].

CKD is a multifaceted syndrome with systemic consequences that extend beyond progressive renal impairment. Patients affected by CKD are at significantly elevated risk for cardiovascular events, mineral and bone disorders and, importantly, infectious complications, which are now recognized as a leading cause of hospitalization and mortality in CKD patients. Recent epidemiological data from the US Renal Data System (USRDS) 2024 Annual Data Report indicate that, while infection-related hospitalization rates have remained relatively stable in the general population, they have increased among patients with advanced CKD, mainly stage 4 and 5 [2].

Infection risk in CKD is multifactorial, involving both intrinsic immune alterations and external exposures such as dialysis procedures and frequent healthcare contact. In this complex landscape, emerging evidence has highlighted the role of the gut microbiome—and specifically the disruption of the so-called gut–kidney axis—as a potential driver of immune dysfunction in CKD [3]. The gut–kidney axis encompasses the bidirectional interaction between renal and intestinal health, mediated through metabolic, immune, and microbial pathways. In the setting of CKD, several factors including uremia, reduced dietary fiber intake, chronic inflammation, and polypharmacy—particularly the use of phosphate binders, iron supplements, and antibiotics—contribute to the disruption of gut homeostasis, leading to gut dysbiosis [4].

This dysbiotic state is characterized by a reduction in beneficial commensals [e.g., short-chain fatty acid-producing bacteria] and an overgrowth of pathogenic species, which can enhance the production of uremic toxins such as indoxyl sulfate (IS) and p-cresyl sulfate (PCS). These toxins, in turn, exert deleterious effects on both the kidney and systemic immunity. Furthermore, dysbiosis has been implicated in compromising the integrity of the intestinal epithelial barrier, increasing intestinal permeability and facilitating the translocation of microbial products into the systemic circulation—a process that can trigger chronic low-grade inflammation and impair host defense mechanisms [5].

Recent studies have proposed that gut dysbiosis may represent a modifiable contributor to the heightened susceptibility to infections observed in patients with CKD. By altering both innate and adaptive immune responses, microbial imbalances may reduce the effectiveness of antimicrobial defenses and vaccination strategies.

Although promising, significant gaps remain in our understanding of the specific mechanisms linking gut dysbiosis to infection risk in CKD, as well as in the long-term clinical evidence supporting efficacy and safety of microbiota-targeted interventions. In this narrative review, we have discussed the current understanding of gut microbiota alterations in CKD, their mechanistic link to infection risk, and discuss emerging therapeutic approaches—including prebiotics, probiotics, synbiotics, and fecal microbiota transplantation—aimed at restoring intestinal eubiosis and mitigating infection-related complications in CKD.

## Gut microbiome alterations in CKD: the gut–kidney axis

The human microbiome encompasses all microbial DNA in the body and resides in multiple sites, including the skin, and—most abundantly—the gut. In particular, the gastrointestinal tract harbors trillions of microorganisms—including bacteria, archaea, fungi, bacteriophages, and eukaryotic viruses—with the majority of bacterial species belonging to two phyla: Firmicutes and Bacteroidetes. These microbes play essential roles in metabolism, immune regulation, and maintenance of gut barrier integrity [6]. Growing clinical and experimental evidences has well established the existence of a bidirectional communication between the gut and the kidneys, commonly referred to as the gut–kidney axis [3].

Gut dysbiosis, a condition characterized by disruption of the microbial balance, has been linked to systemic inflammation and is thought to contribute to the pathogenesis and progression of both acute kidney injury (AKI) and CKD [7].

Multiple factors can drive gut dysbiosis in CKD, often involving complex host–microbiota interactions. Key contributing causes include: dietary restrictions, medication use, and uremia.

CKD dietary management often involves reduction of fruits and vegetables intake due to their high potassium content, as well as restriction of cheese and yogurt due to their high phosphorus content. These dietary limitations reduce the intake of foods with beneficial microbial properties and impair gut motility, thereby contributing to dysbiosis [8].

Patients with CKD are often on polypharmacy due to the burden of multiple comorbidities. Medications such as proton pump inhibitors [PPIs], phosphate binders [9], iron supplements [10], immunosuppressants [11], and antibiotics [12] can significantly alter gut microbiota composition. In particular, prolonged antibiotic use depletes both pathogenic and commensal bacteria, reducing microbial diversity and favoring the growth of antibiotic-resistant strains. As a result, patients may become more susceptible to infections and experience compromised gut epithelial integrity.

Loss of kidney function leads to the accumulation of metabolic waste products, such as urea, in the bloodstream instead of being excreted in the urine. The elevated urea level diffuses into the gut lumen, where urea is hydrolyzed by bacterial urease into ammonium. This alters the gut environment, promoting the overgrowth of urease-producing bacteria and increasing luminal pH, which impairs gut barrier integrity and contributes to dysbiosis [13].

The accumulation of uremic toxins is a hallmark of CKD and significantly accelerates disease progression. It promotes a shift in the gut microbial profile from saccharolytic to proteolytic species, resulting in the production of gut-derived uremic toxins—such as PCS, IS, phenyl sulfate, and trimethylamine-N-oxide [TMAO]—via amino acid catabolism [14]. Several studies have demonstrated the deleterious effects of TMAO on the kidney, including interstitial fibrosis, endothelial dysfunction, decreased eGFR, and an increased risk of both cardiovascular events and infections. Moreover, PCS and IS exhibit high plasma protein binding, which limits their clearance by standard dialysis techniques [15].

**Fig. 1:**
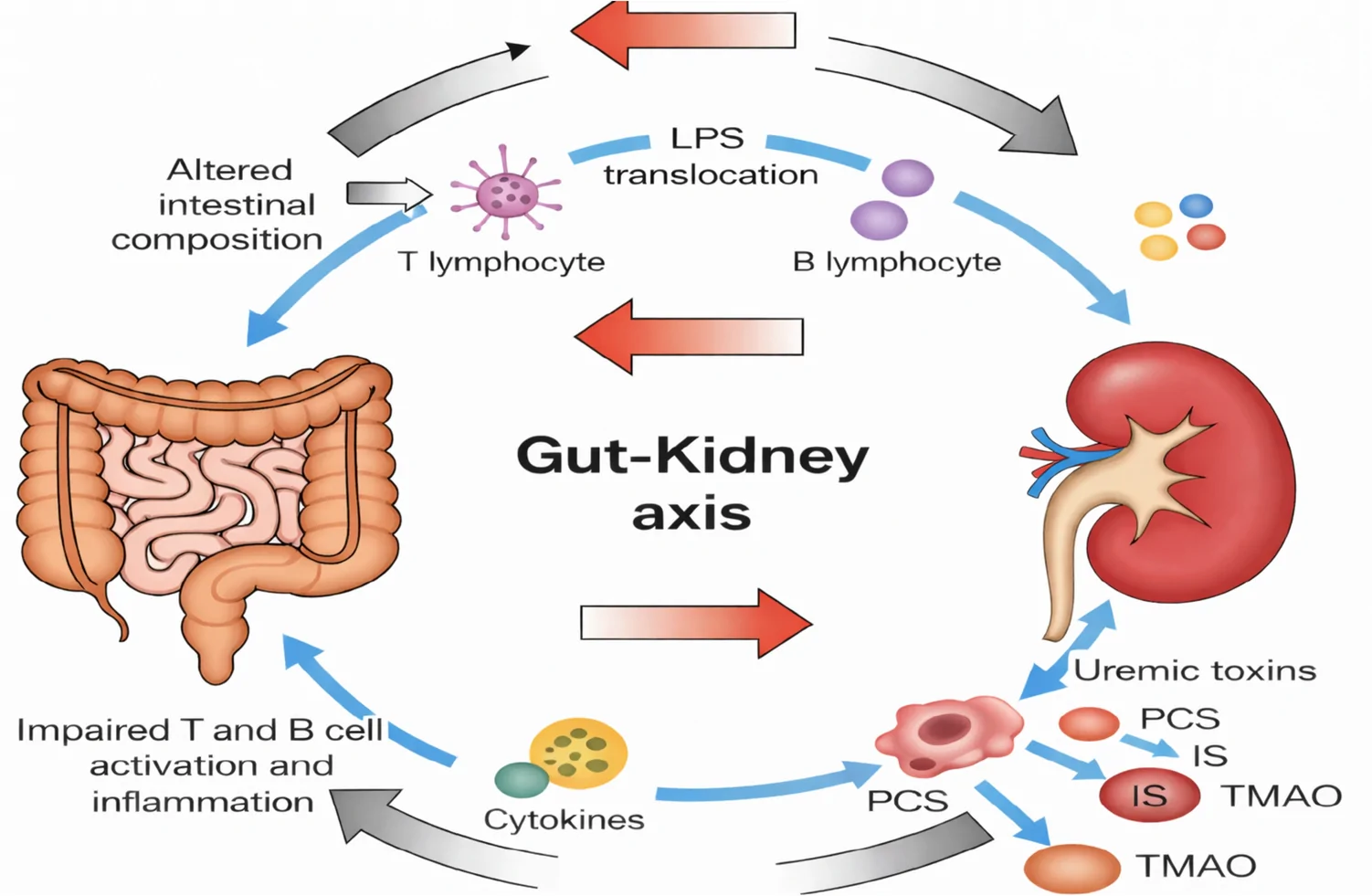
Schematic diagram summarizing the proposed mechanisms related to the gut–kidney axis involved in the pathogenesis of chronic kidney disease and its comorbidities. Cytokines involved in this process may originate from multiple sources, including renal, gut and immune cells. LPS = lipopolysaccharide; IS = indoxyl sulfate; PCS = p-cresyl sulfate; TMAO = trimethylamine-N-oxide. The accumulation of uremic toxins promotes a shift in the gut microbial profile from saccharolytic to proteolytic species, resulting in the production of gut-derived uremic toxins—such as PCS, IS, phenyl sulfate, and TMAO. This facilitates bacterial translocation and the release of pro-inflammatory mediators, which may ultimately promote systemic inflammation and increase infection risk in CKD

Other microbiota-derived metabolites such as short-chain fatty acids [SCFAs]—including acetate, butyrate, and propionate—are also altered in CKD. These are the main energy source for colonic epithelial cells and play a critical role in maintaining gut barrier integrity by modulating tight junction proteins and immune responses. In CKD, the reduction in SCFA production caused by dysbiosis leads to increased luminal pH and compromised barrier function, contributing to the so-called “leaky gut” phenomenon [16]. This facilitates bacterial translocation and the release of pro-inflammatory mediators, which may ultimately promote systemic inflammation and increase infection risk in CKD. This pathological sequence has been recognized as a key mechanism underlying the gut–kidney axis in chronic kidney disease [Fig. 1].

## Linking dysbiosis to increased infection risk

Progress over the past decades has established a strong link between CKD and CKD-related dysbiosis that contributes to infection susceptibility mainly through [a] impaired gut barrier function and bacterial endotoxin translocation, [b] immune system modulation is impaired due to the accumulation of uremic toxins, [c] translocation of microbial DNA fragments, and [d] circulating microbiota as an indicator of infection.

Bacterial endotoxin, a major component of the outer membrane of Gram-negative bacteria, is among the most studied immunostimulatory microbial products [17]. Circulating endotoxins bind to toll-like receptor 4 [TLR-4] on immune cells, particularly monocytes and macrophages, triggering inflammatory responses.

In CKD patients—especially those undergoing dialysis—gut permeability is often compromised. This facilitates the translocation of bacterial endotoxins from the intestinal lumen into systemic circulation, contributing to a pro-inflammatory state and increasing infection risk.

CKD is characterized by increased levels of pro-inflammatory cytokines, particularly tumor necrosis factor-alpha [TNF-α] and interleukin-6 [IL-6], which are strongly associated with disease progression. Moreover, neutrophils and monocytes in CKD patients are hyperactivated and exhibit enhanced sensitivity to lipopolysaccharides [LPS] [18]. This immune activation is partly driven by the accumulation of gut-derived uremic toxins, such as PCS and IS. Notably, IS activates the aryl hydrocarbon receptor in monocytes, promoting the production of pro-inflammatory cytokines and contributing to infection susceptibility [19].

Both innate and adaptative immune responses are affected: uremic toxins reduce neutrophil chemotaxis and oxidative burst, impair dendritic cell function, and lead to defective T and B cell responses [20–22]. The translocation of microbial DNA fragments from the gut into the systemic circulation has been observed in both CKD and ESKD and is associated with elevated levels of inflammatory markers such as C-reactive protein [CRP] and interleukin 6 [IL-6] as reported in several experimental studies involving uremic rats.

Bacterial DNA fragments are consistently detectable and easily discerned from human DNA due to their highly conserved unique 16S ribosomal RNA (rRNA) subunit. Because of this nature of bacterial DNA fragments, they have recently been recognized as a better quantitative marker of circulating bacterial load compared with bacterial endotoxin which enables the detection of only Gram-negative bacteria. Moreover, in patients with CKD, the profiles of circulating16S rRNA are different from those in healthy individuals [23, 24].

Bacterial DNA, recognized by the immune system as foreign due to its molecular structure, is linked to infection and to inflammation. Its presence activates multiple cells signaling pathways, including the mitogen-activated protein kinases and nuclear factor kappa B cascades. In inflammatory cells such as polymorphonuclear leukocytes, bacterial DNA fragments can induce significant biological responses by modulating chemokine expression and phagocytic activity [25, 26].

Emerging evidence suggests that gut microbiota may translocate into the systemic circulation in patients with CKD, contributing to infection risk and immune dysregulation. Studies have started to identify circulating microbiota components as potential biomarkers for disease activity in kidney and infection-related conditions.

In a pilot study of Shah et al., comparing the composition of circulating microbiota between non-diabetic CKD patients without kidney replacement therapy and healthy individuals, patients with CKD (vs. healthy individuals) displayed a significant higher relative abundance of *Enterobacteriaceae and Pseudomonadaceae* families, *Gammaproteobacteria* class, and *Proteobacteria* phylum in their buffy coat samples [27]. Recently, Sumida et al., using serum samples of 34 hemodialysis patients enrolled in a pilot case–control study (17 cases with a fatal cardiovascular event and 17 matched controls without such an event during a median follow-up of 2.0 years) showed that the proportion of *Proteobacteria* and *Actinobacteria* phyla were significantly correlated with blood levels of nuclear factor erythroid 2-related factor 2 (Nrf2), a master regulator of antioxidative responses, and were marginally associated with a greater risk of cardiovascular mortality, independently of age, sex, race, dialysis vintage, and type of vascular access [28].

Of note, this study utilized serum samples to assess the circulating microbiota, focusing on the cell-free fraction of blood. In most previous studies, the circulating microbiota was assessed using leukocyte-containing blood fractions, the nature of which may differ from that of the circulating microbiota identified in the cell-free blood fraction. More specifically, the circulating “cell-free” microbiota located in plasma or serum fraction may exert its potential pathophysiological effects on immune cells (through their receptors [e.g., TLR-9]) in a more direct manner, which in turn suggests the potential of the circulating cell-free microbiota as a more clinically applicable, non-invasive diagnostic/prognostic biomarker compared with the circulating microbiota identified from other leukocyte-containing blood fractions.

However, the current body of literature remains limited, and the association between circulating microbiota composition and clinical outcomes is still under investigation. Furthermore, the precise origin and clinical significance of circulating microbiota in CKD are yet to be fully understood, requiring further research and standardization [27].

Studies on the mechanisms linking dysbiosis to infection are summarized into Table 1**.**

**Table 1:** Mechanisms showing a link between dysbiosis and infection risk

Author/ref.	Population (n)	Study design	Duration	Result
*Bacterial endotoxin translocation*				
Shi k et al.	HD (52)	Single center, prospective	6 months	↑ bacterial translocation and microinflammation in ESKD than hemodialysis
McIntyre CW et al.	CKD stage 3–5 (249)	Single center, retrospective	1 year	↑ endotoxemia in CKD
Andersen K et al.	CKD mice	Experimental study	9 weeks	Uremia is associated with dysbiosis
*Accumulation of uremic toxins*				
Lim WH et al.	HD; PD	Experimental study in vitro	5 days	Uremic toxins inhibit dendritic cells
Shiba T et al.	CKD mice	Experimental study	4 weeks	PCS reduce peripheral B cell in mice with CKD
*Translocation of Microbial DNA fragments*				
Navarro MD et al.	ND CKD stage 5 ; HD (20)	Prospective	undefined	Bacterial DNA prolongs the survival of inflamed mononuclear cells in hemodialysis patients; ↑ of IL 6 And PCR
Merino A et al.	HD (20)	Comparative		Bacterial DNA stimulates activated monocytes
*Circulating microbiota*				
Shah NB et al.	CKD (20)	Cross-sectional		Differences in microbiome between CKD and healthy control

## Targeting the microbiome in CKD: potential interventions

**Fig. 2:**
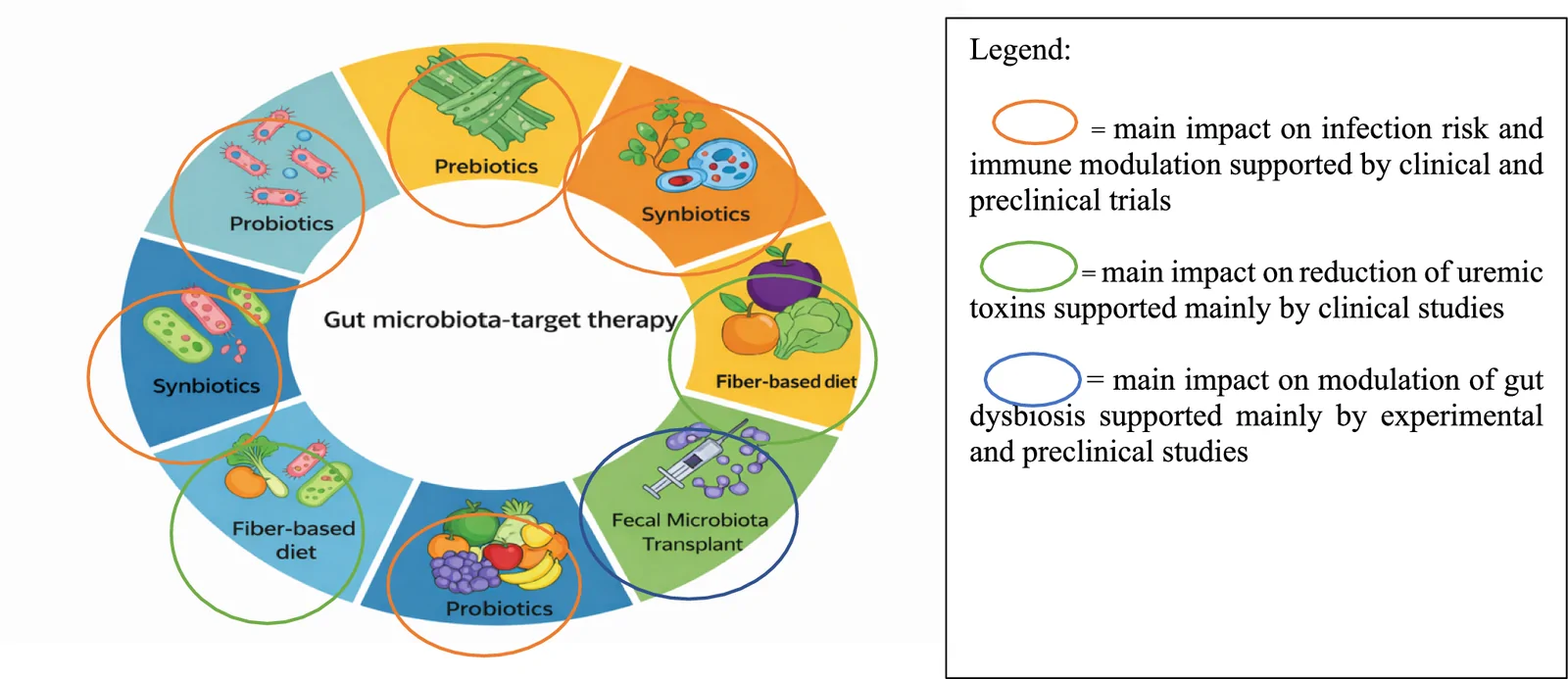
Schematic diagram of the potential gut microbiota-targeted therapy in CKD. The figure summarizes the main microbiota-directed interventions, including dietary approaches, prebiotics, probiotics, synbiotics, and fecal microbiota transplantation. Each intervention is associated with its principal proposed mechanism of action as well as with the level of supporting evidence

Improved understanding of the pathophysiologic roles of gut dysbiosis has led to increasing interest in microbiota-targeting therapeutic strategies aimed at preventing and treating CKD and its infection-related complications. Interventions include dietary modifications [plant-based and low-protein diets], supplementation with prebiotics [nondigestible food ingredients that induce specific modifications in the composition and/or activity of the gut microbiota], probiotics [live microorganisms which confer health beneficial effects when administered in adequate amounts to the host] and synbiotics [both probiotics and prebiotics], constipation management, fecal microbiota transplantation, intestinal dialysis, and exercise-based approaches [28] [Fig. 2].

### Plant-based diet

Plant-based diets emphasize the consumption of plant-derived foods such as whole grains, legumes, seeds, nuts, fruits, and vegetables, while limiting animal-based products such as meat, fish, dairy, and eggs. These diets are rich in dietary fibers—both soluble and insoluble—and contain compounds that beneficially influence gut microbiota composition.

Nondigestible fibers—such as resistant starch, cellulose, and lignin—pass intact into the large intestine, where they are fermented by anaerobic microbes, increasing stool bulk and decreasing intestinal transit time. These processes not only promote regular bowel movements, reducing endotoxin absorption, but also contribute to systemic anti-inflammatory effects [29].

From an epidemiological perspective, the beneficial effects of a plant-based diet in CKD have been reported in several observational studies and clinical trials. In an observational study of 15 vegetarians and 11 individuals consuming an unrestricted diet, the production rates of PCS and ISwere markedly lower in vegetarians than in individuals consuming an unrestricted diet [30]. Using a prospective cohort of 14 686 middle-aged adults in the Atherosclerosis Risk in Communities study, *Kim et al.* showed that higher (vs. lower) adherence to a plant-based diet was significantly associated with lower risk of incident CKD and slower estimated glomerular filtration rate decline [31]. In addition, plant-based diets tend to be lower in saturated fats and richer in antioxidants and phytochemicals, such as polyphenols, that may shift microbial communities toward anti-inflammatory profiles. Epidemiological studies suggest that these diets are associated with lower levels of protein fermentation by gut microbes and reduced production of toxins. They are also linked to lower dietary phosphorus and protein loads, both of which are relevant in CKD management [32].

A recent study of 2025 that evaluated the effect of a diet high in plant diversity in individuals with CKD stages 3–4 reported changes in gut microbiota, including a shift toward increased production of butyrate/isobutyrate, and achieved decreased levels of IS and PCS only in patients with more advanced CKD [33]. Since protein-bound uremic toxins such as PCS and IS have been implicated in immune dysfunction in CKD, their reduction through plant-based dietary patterns may indirectly contribute to improved host defense and lower susceptibility to infections, although direct clinical evidence remains limited. While there are many benefits and enthusiasm to embrace Mediterranean and plant-based diets, there are few clinical trials to examine how these may impact the gut microbiota and, more importantly, health outcomes. This is partly related to safety concerns that increased plant-sourced potassium may contribute to hyperkalemia [33].

Despite concerns regarding hyperkalemia risk, current evidence does not consistently support this in well-managed patients. Increased fecal potassium excretion and the buffering effect of plant fibers may help mitigate this risk. However, comprehensive data on which to base CKD stage-specific dietary recommendations that take the microbiota into account are currently lacking.

### Low-protein diet [LPD]

The major benefits of LPD are potentially attributable to gut microbiota modulation through reduced production of uremic toxins.

There are few studies that support utilizing LPDs as possible gut microbiota-targeted interventions. For example, a recent pre-post study of 16 CKD patients found that a 6-month intervention with an LPD significantly modified gut microbiota and modulated inflammatory and metabolic parameters [34].

In another observational study of 30 NDD-CKD patients undergoing LPD for 6 months, *Black et al.* demonstrated that those who adhere (vs. did not adhere) to the LPD had significantly lower levels of serum PCS, along with the change in the gut microbiome profile [35].Since plant [vs. animal] protein intake has been associated with lower risk of incident CKD, the source of protein, as well as its amount, may need to be considered for the implementation of an LPD in patients with CKD. In this context, a plant-dominant LPD is associated with both infection control and kidney health [35, 36].

### Supplementation of prebiotics, probiotics, and synbiotics

The dietary supplementation of prebiotics, probiotics, and synbiotics has been increasingly recognized as a potential gut microbiota-targeted intervention for patients with CKD and World Health Organization [WHO]-defined probiotics as “live microorganisms which when administered in adequate amounts confer a health benefit on the host”[37].

Several intestinal bacteria such as *Lactobacillus*, *Bifidobacterium,* and *Enterococcus* are beneficial and help in improving the stability of gut microbiota. Probiotics are helpful for human health in several aspects and in particular as antimicrobials and immunity stimulation through the inhibition of other pathogens by producing several inhibitory substances such as organic acids, short-chain fatty acids, carbon dioxide, hydrogen peroxide, acetaldehyde, acetoin, and diacetyl [38–40].

Other mechanisms of probiotics action are competitive inhibition on intestinal epithelial surface by blocking the adhering sites of the intestinal epithelial surface, reduction in the pathogen’s interaction, and inhibition of the pathogens that consume the nutrients and deprive the host [41–44].

Several meta-analyses of the impact of probiotics on CKD indicate probiotics may also reduce PCS and increase interleukin-6 levels and facilitate improvements in gastrointestinal symptoms [45]. However, there are still unanswered questions regarding how probiotics in CKD can improve long-term health outcomes.

Prebiotics, instead, work by providing energy sources for gut microbiota or are fermented into substrates that feed the growth of other microorganisms [46]. Inulin, fructooligosaccharides, galactooligosaccharides, soy oligosaccharides, xylooligosaccharides, and pyrodextrins are commonly used as prebiotics [47]. They promote the growth of *Bifidobacteria* and *Lactobacilli* species, while suppressing other groups of bacteria such as *Bacteroides*, *Clostridia*, and *Enterobacteria* species [48]. Clinical studies evaluating prebiotics in CKD patients show, in fact, promising results in improving gut microbiota composition and reducing inflammatory markers and uremic toxins. For example, inulin-type prebiotics are associated with increased levels of beneficial *Bifidobacterium* and *Faecalibacterium prausnitzii*, known for their anti-inflammatory effects. Galacto-oligosaccharides have been found to increase beneficial gut bacteria, indirectly influencing the abundance of potentially pathogenic bacteria through increased production of SCFA and lactic acid, which may lower luminal pH, inhibit the growth of certain pathogens, and support mucosal barrier function [49]. Synbiotics are the combination regimen of prebiotics and probiotics. Recently, it has been reported that the use of synbiotics has significantly decreased several cardiovascular risk factors and inflammatory risk markers; however direct effects on infection have not been specifically demonstrated. [50–52].

Trials of synbiotic supplementation for people with CKD are under development. Rossi et al. explored synbiotic supplementation for 6 weeks with nine strains from the *Lactobacillus*, *Bifidobacteria,* and *Streptococcus* genera with 15 g of galactooligosaccharide, inulin, and fructooligosaccharide. Synbiotic therapy was associated with increased Bifidobacterium genera and reduced the *Ruminococcaceae* family. However, there were increases in albuminuria. The same group has conducted follow-up studies on the long-term feasibility of synbiotics [51].

McFarlane et al. used 20 g of resistant starch fiber and the same probiotic species and found enrichments in acetate- and butyrate-forming species (*Bifidobacterium* and *Blautia*) [51].

Unfortunately, there is insufficient evidence to conclude whether one type of supplementation is superior to another. Future well-designed clinical trials are needed to establish the appropriate microbiota-targeted supplementation formulation and to confirm its effectiveness on clinical outcomes. Table 2 shows the major studies and results related to the use of prebiotics, probiotics, and synbiotics in the treatment of gut dysbiosis.

**Table 2:** Therapeutic interventions with prebiotics, probiotics, and synbiotics and related studies

Author/ref	Population (n)	Intervention	Study design	Duration	Result
*Probiotics*					
Hida et al.	HD (25)	*B. infantis, L. acidophilus, E. faecalis*	Single-center, observational trial	4 weeks	↓ Indican in feces and serum and ↓ p-cresol in feces
Simenhoff et al.	HD (8)	*L. acidophilus*	Single-center, observational trial	One course	↓Dimethylamine ↓ nitrosodim-ethylamine
Takayama et al	HD (22)	*B. longum strain*	Single-center, non-randomized, placebo-controlled trial	5 weeks	↓ IS
Taki et al.	HD (27)	*B. longum*	Single-center, non-randomized, placebo-controlled trial	3 months	↓Homocysteine, IS, and triglycerides
Natarajan et al.	HD (22)	*S. thermophilus, L. acidophilus, B. longum*	Single-center, double-blind, placebo-controlled, randomized crossover trial	2 months	Quality of life, trend ↓ of serum indoxyl glucuronide and C-reactive protein
Wang et al	PD (29)	*B.bifidum, B.catenulatum, B. longum, L. plantarum*	Single-center, double-blind, placebo-controlled, randomized trial	6 months	Serum TNF-α, IL-5, IL-6, and LPS; preservation of residual renal function
Ranganathan et al.	CKD stage 3–4 (46)	*L. acidophilus KB31, S. thermophilus KB27, and B. longum KB35*	Multicenter, prospective, randomized, double-blind, crossover, placebo-controlled trial	6 months	↓ BUN ↑ Quality of life
Mafi et al.	ND CKD (DM) (60)	*Lactobacillus acidophilus ZT-L1, Bifidobacterium bifidum ZT-B1, Lactobacillus reuteri ZTLre, Lactobacillus fermentum ZT-L3*	Randomized, placebo-controlled trial	12 weeks	↓ Fasting glucose ↑ Serum insulin ↓HOMA-IR ↓ CRP ↑ HDL cholesterol ↑Total glutathione ↑Plasma betaine
*Prebiotics*					
Xie et al.	HD (124)	Dietary fiber	Randomized placebo-controlled trial	6 weeks	↓Total and LDL cholesterol; plasma IL-6, IL-8, and Hs-CRP ↑ Total antioxidant capacity (T-AOC)
Esgalhado et al.	HD (31)	Resistant starch	Randomized, double-blind, placebo-controlled trial	4 weeks	↓ IL-6 ↓ IS ↓ TBARS
Laffin et al.	HD (20)	Resistant starch	Randomized, double-blind, placebo-controlled trial	2 months	↓ BUN ↓ IL-6, TNF- ↑αFaecalibacterium
*Synbiotics*					
Viramontes-Horner et al.	HD (42)	*L. acidophilus and B. Lactis + inulin*	Multicenter, double-blind, placebo-controlled, randomized, clinical trial	2 months	Safe; improved gastrointestinal symptoms; Trend to ↓ plasma C-reactive protein levels
Guida et al.	CKD stage 3–4 (30)	*L. plantarum, L. casei subsp. rhamnosus, L. gasseri, B. infantis, B. longum, L. acidophilus, L. salivarius, L. sporogenes, and S. thermophilus + prebiotic (inulin and tapioca-resistant starch)*	Single-center, double-blind, placebo-controlled, randomized crossover trial	4 weeks	↓ Plasma p-cresol
Rossi et al.	CKD stage 4–5 (37)	*Lactobacillus, Bifidobacteria, and Streptococcus genera + inulin, fructooligosaccarides, and galacto-oligosaccarides*	Single-center, double-blind, placebo-controlled, randomized crossover trial	6 weeks, with a 4-week washout before crossover	No change in serum IS, ↓ Serum PCS; ↑ Bifidobacterium and ↓ Ruminococcaceae; ↑ Albuminuria

### Constipation treatment

Constipation is one of the most common gastrointestinal disorders among patients with CKD, partly because of their sedentary lifestyle, low fiber and fluid intake, multiple comorbidities, and concomitant medications [8].

Although constipation is usually perceived as a benign self-limited condition, emerging evidence indicates its independent association with adverse clinical outcomes, such as ESKD and mortality, suggesting a greater importance of the adequate management of constipation [53].

In patients with constipation, dietary modification with increased dietary or supplemental fiber intake is traditionally considered a first-line non-pharmacological therapeutic option.

Although patients with CKD are typically advised to restrict the intake of fiber-rich foods to prevent hyperkalemia, given the lack of evidence supporting a plant-based diet, the potential gastrointestinal benefits of dietary fiber, along with its low cost, may justify consideration of a fiber-rich diet as a first step in the treatment of constipation in CKD.

However, pharmacological interventions with laxatives are often required to treat constipation in patients with CKD, particularly in its advanced stages and in patients with hyperkaliemia, in order to manage also this alteration [54].

### Fecal microbiota transplantation

Fecal microbiota transplantation [FMT] is a method of transferring the gut microbiota from healthy individuals to patients with gut dysbiosis [typically through an oral administration of encapsulated formulations] [55].

Although there is currently no trial evidence that supports the clinical utility of FMT in the CKD population, the successful clinical application of FMT in treating patients with constipation may support the continued exploration of this therapeutic potential for CKD. Today, FMT is recognized as a clinically highly effective treatment for recurrent *Clostridioides* difficile infection, but FMT is also explored for other indications.

Initial FMT applications have shown promise. For example, in the PREMIX trial, 8 of 9 renal transplant recipients with enteric multidrug-resistant bacterial colonization had negative stool cultures after FMT treatment, and key taxonomic and metabolite dynamics were associated with response to treatment [56, 57]. Additional translational studies of FMTs and consortia of bacteria associated with positive responses are needed to expand microbiota treatment options for patients with kidney disease.

### Colonic adsorbents

Agents like activated charcoal [e.g., AST-120] have been shown in small human and animal CKD studies to markedly reduce oxidative stress, proteinuria, and inflammation. In addition, using AST-120 reduced the concentrations of IS and p-CS in animal models. Powered studies are required to evaluate the benefits of health outcomes in CKD patients [58].

### Antimicrobial stewardship [AMS]

Antimicrobial stewardship [AMS] programs are structured, evidence-based initiatives aimed at optimizing antimicrobial prescribing practices through the careful selection of the most appropriate antibiotic agent, dosing regimen, duration of therapy, and route of administration. In patients with CKD, AMS programs are particularly relevant due to their dual objective of minimizing both antimicrobial resistance and gut microbiota disruption—two critical concerns in this vulnerable population [59]. CKD patients are frequently exposed to antibiotics owing to their increased susceptibility to infections, repeated hospitalizations, and the presence of invasive devices such as dialysis catheters. This repeated exposure poses a substantial risk for the development of multidrug-resistant organisms and the perturbation of gut microbial homeostasis.

Multiple studies have demonstrated that antibiotics—particularly broad-spectrum agents—exert a profound impact on the composition and metabolic function of the gut microbiota [60]. Antibiotic-induced dysbiosis is characterized by a reduction in microbial diversity, depletion of beneficial commensal taxa, and overgrowth of potentially pathogenic and resistant strains [61]. In patients with CKD, such alterations may be especially detrimental, as they further compromise mucosal barrier integrity and immune competence, thereby increasing the risk of bacterial translocation, systemic inflammation, and recurrent infections. Moreover, this dysbiotic environment may amplify the production and systemic absorption of gut-derived uremic toxins, contributing to the progression of renal dysfunction and associated complications.

Several large meta-analyses have supported that antimicrobial stewardship programs (ASPs) lead to a decrease in infections with MDROs, while others found the correlation inconclusive [61, 62]. One large meta-analysis concluded that ASPs are successful in reducing MDRO colonization, independently of infection, and this success was higher when ASPs were combined with good infection control protocols [63]. AMS programs in nephrology settings should incorporate tailored strategies for CKD patients, including renal function-adjusted antibiotic dosing, early de-escalation guided by microbiological data, and stringent avoidance of unnecessary antibiotic use. Additionally, AMS efforts should be aligned with interventions that support microbiota preservation, such as the concurrent use of probiotics or dietary modulation. Ultimately, integrating AMS principles into the comprehensive care of CKD patients represents a key opportunity to reduce infectious complications, limit antimicrobial resistance, and mitigate the deleterious consequences of gut dysbiosis on kidney and overall health.

## Conclusions

The connections between the gut microbiota and the kidney epitomizes a complex and dynamic relationship that is essential for preserving the metabolic, immunologic, and inflammatory equilibrium in the human host. In the context of CKD, this balance is profoundly disturbed. The accumulation of uremic toxins, such as indoxyl sulfate and p-cresyl sulfate, the increased translocation of endotoxins like lipopolysaccharides [LPS], the loss of intestinal epithelial barrier integrity, and the dysregulation of both innate and adaptive immunity contribute to a bidirectional pathological process. CKD promotes gut microbial alterations—commonly referred to as dysbiosis—and, in turn, this dysbiotic state exacerbates systemic inflammation, accelerates CKD progression, and increases the risk of infections and other complications. This mutual deterioration establishes a reciprocally reinforcing, deleterious cycle, in which both organs negatively influence each other.

Given this pathophysiological framework, clinical strategies aimed at reestablishing gut–kidney symbiosis have gained considerable interest. These interventions include dietary modifications—particularly those aimed at increasing the intake of fermentable fibers—alongside supplementation with prebiotics, probiotics, and synbiotics. More advanced and investigational approaches, such as AMS programs tailored to reduce antibiotic-induced dysbiosis and fecal microbiota transplantation [FMT], have also emerged as promising modalities. These strategies aim to reduce the burden of uremic toxins, restore eubiosis, improve intestinal barrier function, and modulate systemic immune responses.

Future strategies aim to target gut microbial enzymes. For example, the development of oral agents that potently inhibit CutC/D enzymes — which are responsible for the production of TMA by microorganisms — reduced circulating TMAO levels and ameliorated thrombosis risk in a mouse model of carotid artery thrombosis, providing a promising therapeutic treatment also for CKD [64], probably influencing also the levels of inflammatory markers. Our understanding of the role of the gut microbiome across all stages of CKD is still limited by the lack of large, well-characterized population-based cohorts with integrated clinical and microbiome data. Addressing this gap will be essential to clarify how microbiota-targeted interventions may influence immune dysfunction and susceptibility to infections in CKD. In addition, microorganisms with selected or engineered activities represent an exciting therapeutic avenue; however, evidence regarding their impact on infection risk remains scarce and requires dedicated investigation.

Investment in genetic platforms for the rapid manipulation of gut microorganism functions would help to advance research in this area toward clinical translation [65].

However, while preclinical and preliminary clinical studies have yielded encouraging findings, the long-term safety, efficacy, and clinical applicability of these interventions remain to be definitively established. Well-designed, large-scale randomized controlled trials are urgently needed to validate these approaches, optimize their implementation, and determine their capacity to improve clinical outcomes, delay CKD progression, reduce infectious morbidity, and ultimately enhance survival and quality of life in patients with CKD.

## Data Availability

Not applicable.
